# THE RELATIONSHIP BETWEEN BIOGRAPHY, EDUCATION, AND ICT USE IN AGE: A LIFE COURSE PERSPECTIVE

**DOI:** 10.1093/geroni/igae098.3117

**Published:** 2024-12-31

**Authors:** Christina Klank, Tjard de Vries, Ines Himmelsbach, Michael Doh

**Affiliations:** Catholic University Freiburg, Freiburg, Baden-Wurttemberg, Germany; Catholic University Freiburg, Freiburg, Baden-Wurttemberg, Germany; Catholic University Freiburg, Freiburg, Baden-Wurttemberg, Germany; Catholic University Freiburg, Freiburg im Breisgau, Baden-Wurttemberg, Germany

## Abstract

The digital transformation of society is challenging for many older adults, especially those living in institutionalized settings. Support systems are needed, but often lacking. This study first presents a learner-centered approach that focuses on individual interests and abilities of the target group: older adults in institutionalized settings. In order to sustain ICT use (information and communication technology), professionals need to develop programs based on the needs of the target group. Therefore, this study secondly uses a narrative approach to analyze biographies of older adults in order to exploit their potential for conclusions about ICT use. An interdisciplinary German research project established a participatory design for the target group. This design pairs advanced peers with beginners to introduce ICT (e.g. tablets, Internet) based on beginners’ interests and needs. Advanced peers were recruited as volunteers and participated in a train-the-trainer program beforehand. In this study, biographical-narrative and problem-centered interviews were conducted with 42 older German adults (White; age=62-95; 57% female; beginners=24, advanced=18). The interviews were analyzed within a narrative approach to compare subjective narratives about biography, education, and ICT use. Different biographical patterns were identified and are illustrated by two contrasting cases from each group. The results provide evidence that older adults’ biographies influence and partially predict older adults’ motivation and educational interests, which in turn influence ICT use. Additionally, ICT is perceived as either a lifestyle (e.g. meeting communication needs) or a tool (e.g. maintaining independence). When developing educational activities for older adults, we recommend including biographical components in curricula.

